# Yellow Nail Syndrome Associated With a Dental Abscess: A Case Report

**DOI:** 10.7759/cureus.81841

**Published:** 2025-04-07

**Authors:** Shatha Mallah, Haneen Mallah, Fahed Owda, Diana Gomez-Manjarres, Sriram Peruvemba

**Affiliations:** 1 Internal Medicine, An-Najah National University, Nablus, PSE; 2 Pulmonary and Critical Care Medicine, University of Florida Health, Gainesville, USA; 3 Pulmonary, Critical Care and Sleep Medicine, University of Florida Health, Gainesville, USA

**Keywords:** chronic rhinosinusitis, dental abscess, lymphedema, xanthochromia, yellow nail syndrome

## Abstract

Yellow nail syndrome (YNS) is a rare disorder marked by a triad of symptoms: lymphedema, yellow discolored nails, and respiratory manifestations. It is often associated with conditions such as chronic cough, bronchiectasis, pleural effusion, and chronic rhinosinusitis. Although its precise etiology remains elusive, it is believed that impaired lymphatic drainage plays a central role in its pathogenesis. YNS predominantly affects individuals over the age of 50, and there is no definitive treatment, though some cases may achieve partial or spontaneous remission.

In this report, we present the case of a man in his mid-30s with a one-year history of persistent cough, sinus congestion, yellow deformed nails, and lymphedema. Extensive investigations, including imaging and pulmonary function tests, revealed no significant pulmonary abnormalities. The patient underwent multiple rounds of treatments, including antibiotics, antifungal therapy, inhaled and systemic corticosteroids, and nail care regimens, all of which proved ineffective in resolving his symptoms.

Further evaluation, including dental imaging, uncovered an odontogenic abscess. After the infected tooth was extracted, the patient experienced a significant and gradual resolution of his symptoms, including yellow nail discoloration and sinus issues. This case highlights the importance of considering secondary causes, such as dental infections, in the workup of patients with YNS, particularly when standard treatments fail to produce improvements. To our knowledge, this is the first reported case identifying a tooth abscess as the potential cause of YNS.

YNS has been linked to several systemic conditions, including autoimmune disorders, thyroid dysfunction, nephrotic syndrome, and malignancies, but an association with dental abscesses has not been previously documented. This case underscores the critical need for a thorough evaluation of potential contributory factors in YNS, especially in younger patients who do not fit the typical demographic profile. It also emphasizes the importance of timely management of secondary infections, which can lead to significant clinical improvement in such cases. By illustrating a rare presentation of YNS, this case provides further insight into the syndrome's complexity and the potential role of odontogenic infections in its development.

## Introduction

Yellow nail syndrome (YNS) is a rare disorder characterized by a triad of lymphedema, yellow deformed nails, and respiratory manifestations [1]. Several respiratory conditions are associated with YNS, including chronic cough, bronchiectasis, pleural effusion, and chronic rhinosinusitis. Typically, this syndrome manifests in individuals over the age of 50 [2]. The etiology and pathogenesis remain unclear, although it is suspected to be caused by an impaired lymphatic system [3].

There are currently no established guidelines for managing patients with YNS due to the lack of large-scale studies [2]. As a result, treatment primarily relies on anecdotal evidence. Previous case reports and small studies have explored several treatment options, including topical vitamin E and triamcinolone, oral vitamin E and zinc, oral triazole antifungals such as fluconazole combined with oral vitamin E, and oral antibiotics such as clarithromycin. For patients with recurrent pleural effusions, octreotide has been used. Respiratory symptoms, such as sinusitis and bronchiectasis, are typically managed with antibiotics and bronchodilators [4]. Even though the mentioned treatments have achieved partial or complete remission, there is still no definitive cure for YNS [5]. We report a case of a young male with YNS whose condition improved following the drainage of an incidentally discovered odontogenic abscess, despite multiple prior unsuccessful interventions.

## Case presentation

A man in his mid-30s with a history of hypertension (HTN) presented to the Pulmonary Clinic with a year-long history of persistent cough, sinus congestion, thickened yellow nails, and lymphedema. His symptoms began a year ago, initially marked by significant sinus congestion, bilateral yellow nasal drainage, and a persistent cough. Despite undergoing several treatments, including cetirizine 10 mg daily, montelukast 10 mg daily, inhaled and systemic steroids, and neti pot rinses, none of these were effective. He also received multiple rounds of antibiotics, including amoxicillin-clavulanic acid 875-125 mg twice daily for 14 days, doxycycline 100 mg twice daily for 10 days, and ciprofloxacin 500 mg twice daily for 10 days. The only temporary improvement was noted in his sinusitis while on levofloxacin. The patient was diagnosed with HTN at age 17 and has been managed with atenolol-chlorthalidone 50-25 mg daily and lisinopril 10 mg daily. He also reported experiencing lower extremity edema over the past few years.

In addition, he noticed yellow discoloration of his fingernails and toenails for the past eight months, along with a lack of nail growth, which started before taking any prescribed antibiotics. A nail plate punch biopsy performed by the dermatology department revealed Pseudomonas, with clippings testing positive for Grocott’s methenamine silver stain and fungal cultures growing a single colony of Fusarium species.

Despite treatment with oral fluconazole for six months, 1000 units of vitamin E daily, and 140 mg of zinc sulfate twice daily, his condition did not improve. He was also advised to soak his nails in a bleach and vinegar solution with no success. Additionally, he was referred to physical therapy for the management of his lymphedema.

**Figure 1:**
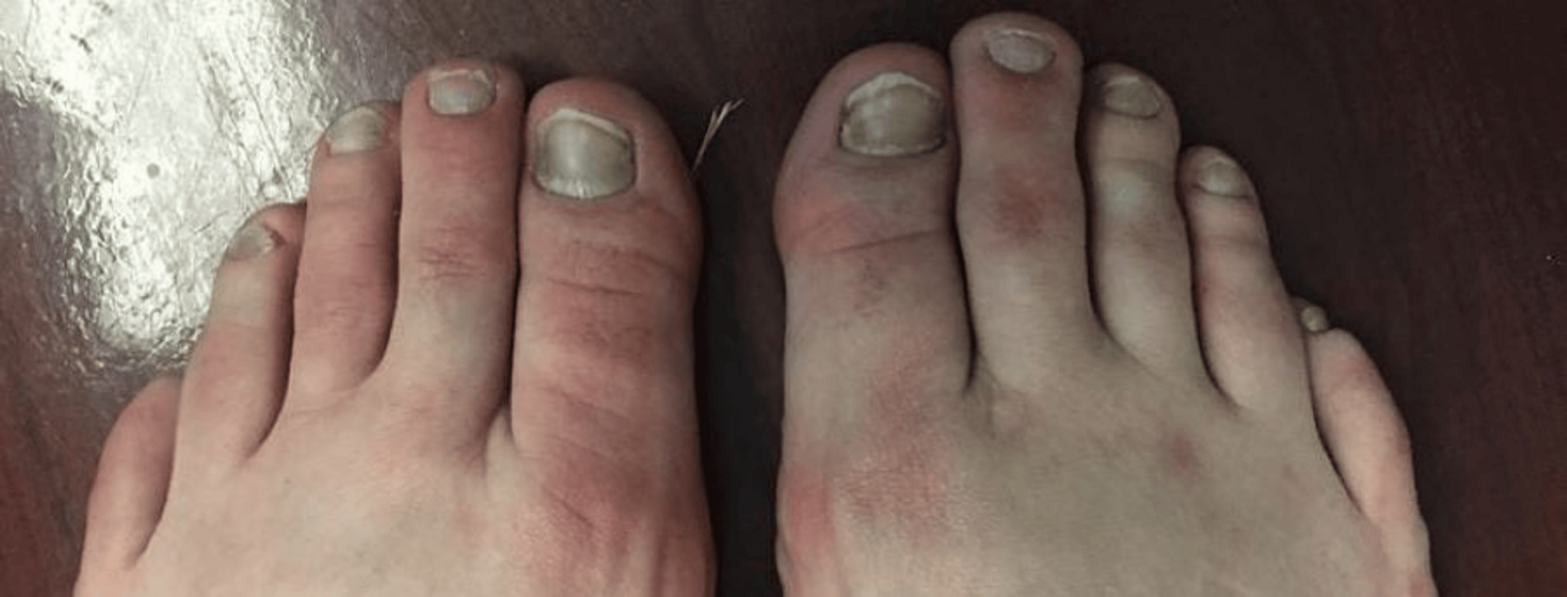
Yellow discoloration of the toenails bilaterally with loss of cuticle and lunula, as well as evidence of paronychia, onycholysis, and erythematous periungual patches

**Figure 2:**
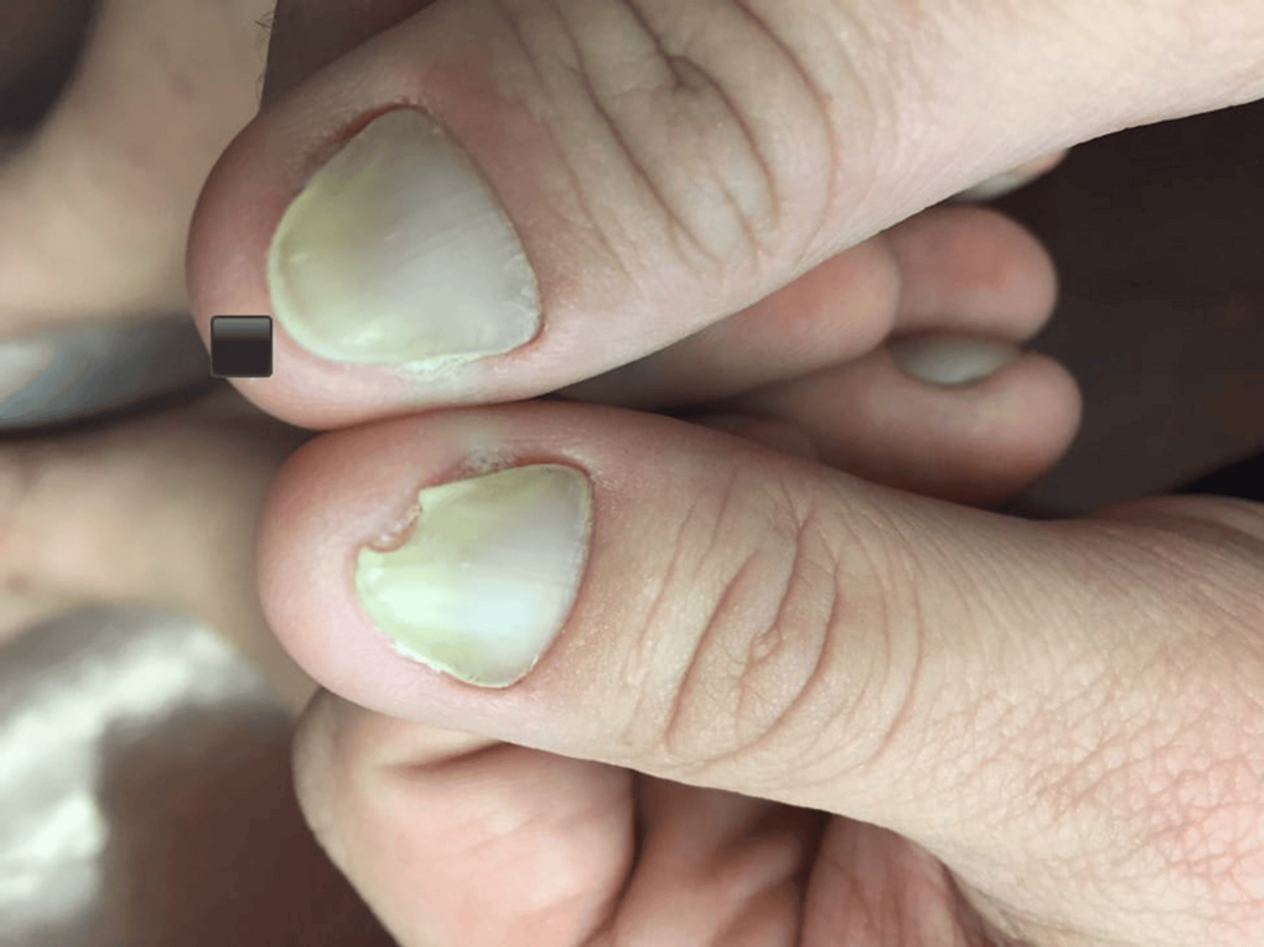
Yellow discoloration of the thumbnails bilaterally with loss of cuticle and lunula, as well as evidence of paronychia, onycholysis, and erythematous periungual patches

On physical examination, his nails exhibited a yellowish discoloration with loss of cuticle (thin layer of clear skin found at the nail bed) and lunula (crescent-shaped whitish area at the base of the nail). Additionally, there was evidence of paronychia, onycholysis, and erythematous periungual patches (Figures 1, 2). He also had lymphedema with a positive Stemmer's sign.

**Figure 3:**
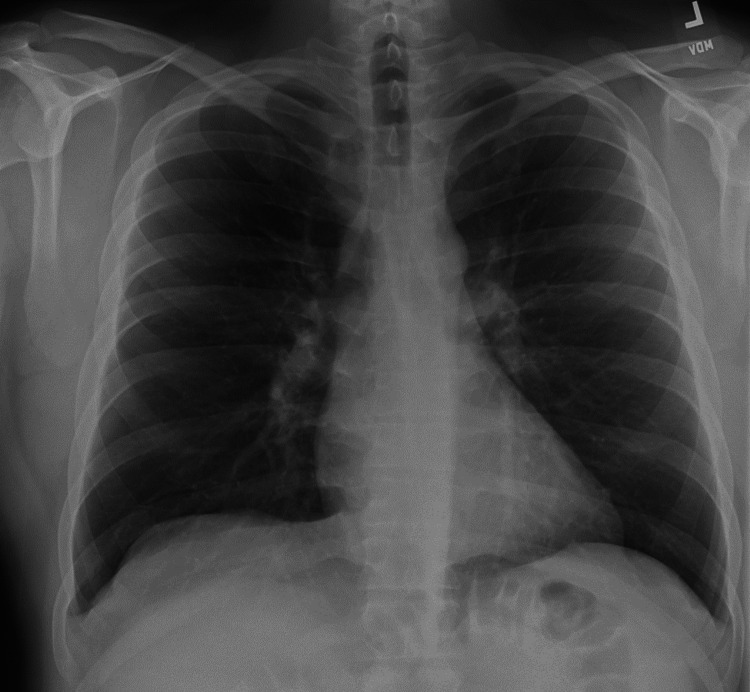
Chest X-ray posteroanterior with no cardiopulmonary abnormalities

**Figure 4:**
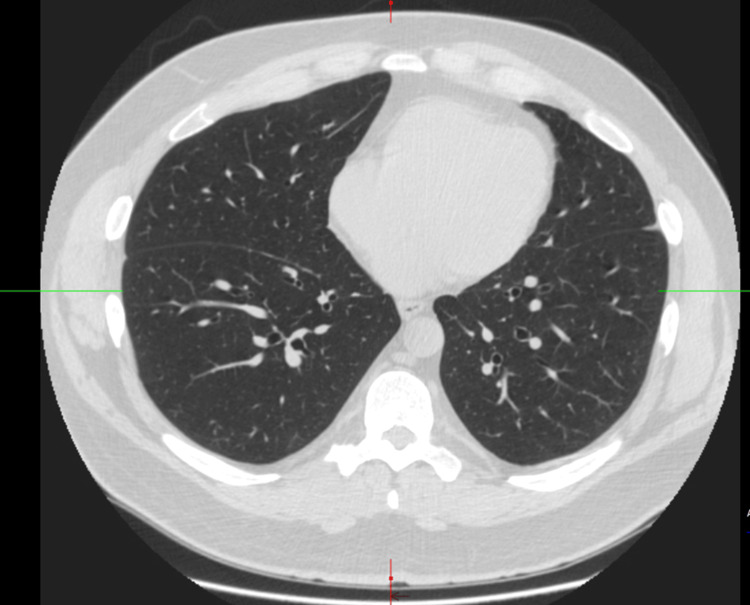
High-resolution CT scan of the lungs with normal parenchyma, and no emphysema or pleural effusion

Labs showed normal immunoglobulins, electrolytes, liver enzymes, kidney function, and autoimmune workup (Table 1). Chest X-ray was normal (Figure 3). A chest high-resolution computed tomography (CT) scan showed no evidence of interstitial lung disease, infiltrate, emphysema, or bronchiectasis (Figure 4). His pulmonary function tests revealed normal spirometry, lung volumes, and diffusion capacity of carbon monoxide.

**Table 1:** Laboratory testing including serum basic metabolic panel, liver enzymes, immunoglobulin levels, autoimmune antibodies, antifungal antibodies, and urinalysis AST: aspartate aminotransferase; ALT: alanine transaminase; ALP: alkaline phosphatase; CK: creatine kinase; CRP: C-reactive protein; Ig: immunoglobulin; CCP: cyclic citrullinated peptide; dsDNA: double-stranded DNA; RF: rheumatoid factor; SS: Sjögren syndrome; RP: RNA polymerase; SRP: signal recognition particle; SNRNP: small nuclear ribonucleoprotein; CENP: centromere protein; PH: potential of hydrogen; WBC: white blood cells

Test	Values
General	
Serum sodium	141
Serum potassium	3.7
Serum chloride	106
Serum calcium	10
Urea nitrogen	16
Serum creatinine	0.93
Serum glucose	88
Total protein	6.6
Serum albumin	4.2
AST	21
ALT	30
Total bilirubin	0.7
ALP	66
Aldolase	5.2
Total CK	99
CRP	3.3
Immunoproteins	
IgM	37
IgG	830
IgA	129
IgE	11
Autoimmune antibodies	
Anti-CCP	<16
Anti-dsDNA	<1
Anti-JO-1	<1
RF	<14
Scl-70	<11
Proteinase-3	<1
SS-A	<1
SS-B	<1
Myeloperoxidase	<1
KU	Not detected
OJ	Not detected
MI-2	Not detected
Scl-100	<11
Scl-75	<11
RP-11	<11
RP-155	<11
SRP	Not detected
U1 SNRNP RNP 70 KD	<11
U1 SNRNP RNP A	<11
U1 SNRNP RNP C	<11
EJ	Not detected
CENP A	<11
CENP B	<11
Fibrillarin	<11
Fungal antibody testing	
Aspergillus fumigatus	Negative
Micropolyspora faeni	Negative
Saccharomonospora viridis	Negative
Urinalysis	
Color	Yellow
Clarity	Slightly cloudy
PH	7
Specific gravity	1.020
Bilirubin	Negative
Blood	Negative
Glucose	Negative
Nitrite	Negative
Leukocyte esterase	Negative
Protein	Trace
WBC	0
Ketones	Negative
Protein/creatinine ratio	0.05

The patient, after a year of the development of his initial symptoms, was diagnosed with YNS of indeterminate etiology and chronic rhinosinusitis. A maxillofacial CT and ENT evaluation were pursued. Maxillofacial CT revealed mucosal thickening in the maxillary, sphenoid, and ethmoid sinuses but no periodontal lucency. A nasal endoscopy demonstrated a thin, white mucous discharge of the middle meatus. He was prescribed amoxicillin-clavulanic acid 875-125 mg twice daily for 21 days, with only temporary improvement in symptoms.

**Figure 5:**
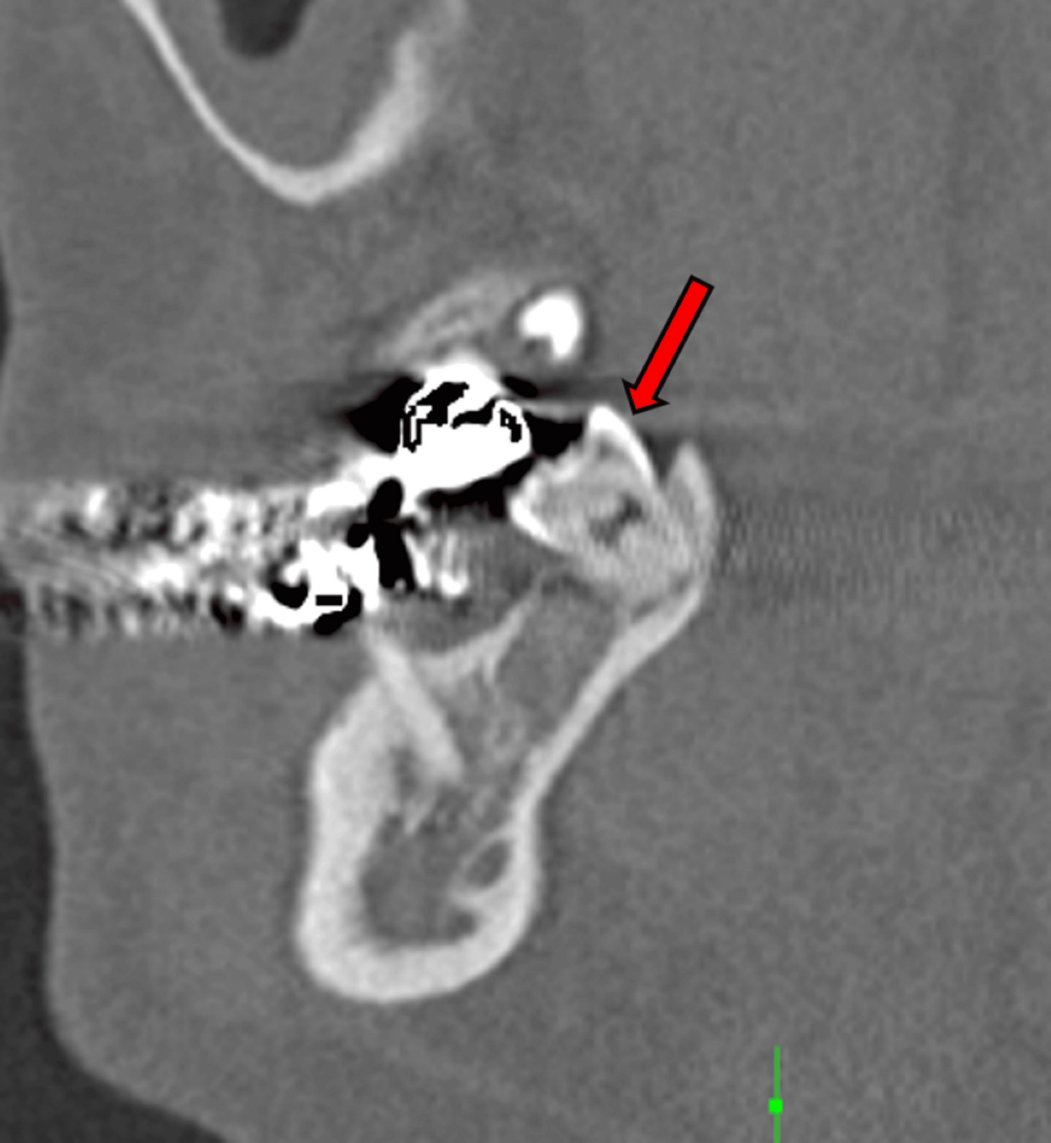
Periapical lucency in the right mandibular tooth (red arrow)

**Figure 6:**
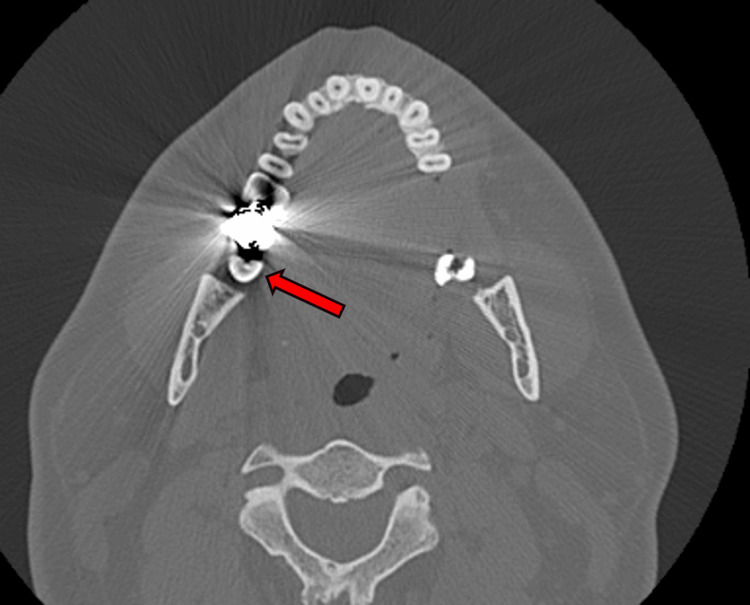
Right mandibular tooth lucency (red arrow)

The patient followed up with an outside ENT a year after his first presentation to our clinic: Repeat sinus CT revealed moderate to marked frothy lobulated opacification of the maxillary, sphenoid, and ethmoid sinuses. A small periapical lucency surrounding the area of the right-sided mandibular tooth (Figures 5, 6) was noted, as well as a right-sided impacted third molar tooth, carious teeth, and a left broken maxillary tooth with a periapical lucency.

**Figure 7:**
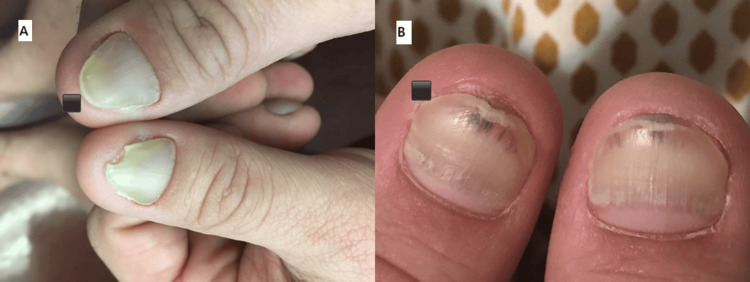
Bilateral thumbnails (a) before and (b) after tooth extraction with clear demonstration of the improvement of the yellow nail discoloration and normal new nail growth

**Figure 8:**
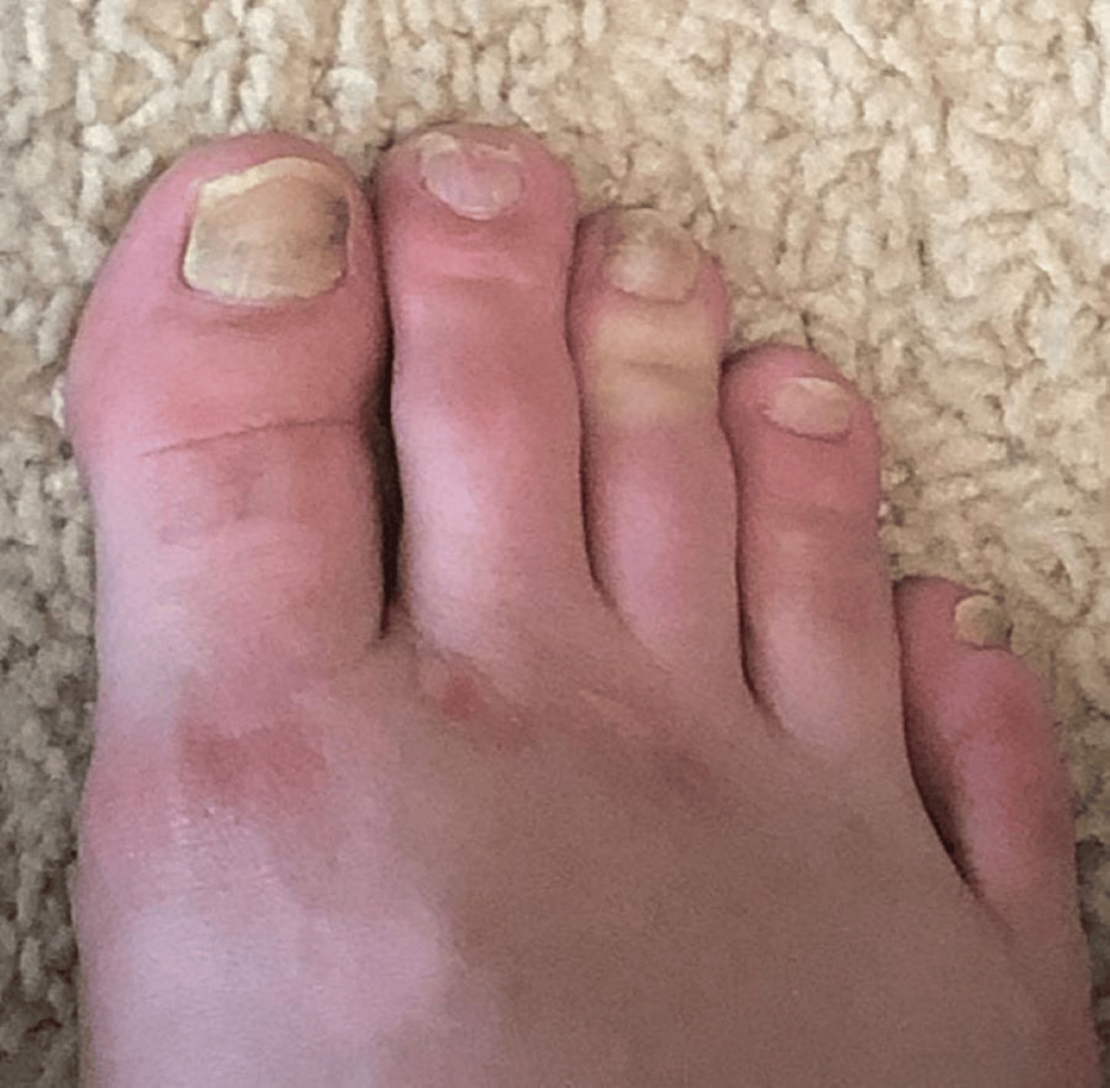
Right foot toenails following tooth extraction with improvement in the yellow discoloration

On examination, mucopurulent drainage was observed from the right middle meatus and right sphenoid. The patient was suspected of having an anaerobic infection of odontogenic origin despite the absence of any oral symptoms. A referral for a dental evaluation revealed an odontogenic abscess, leading to the extraction of the affected right mandibular tooth nearly two years after the onset of symptoms. Following the extraction, the patient was prescribed amoxicillin-clavulanate. Remarkably, the patient's symptoms gradually improved, starting with the resolution of sinusitis. Over time, his nails gradually returned to their normal color, and complete improvement was achieved within three months after the extraction (Figures 7, 8).

## Discussion

YNS is an acquired condition with no sex predominance [6]. This disorder is rare, with an estimated global prevalence of less than one per one million individuals [5]. A defining feature of YNS is its triad of symptoms, including lymphedema, yellowed and distorted nails, and respiratory involvement. According to a study, the complete triad is observed in approximately 60% of affected patients [2].

The hallmark feature of YNS is xanthonychia, the yellow discoloration of nails, which typically presents after the initial onset of other clinical symptoms, including lymphedema and respiratory symptoms [5]. Our case aligns with this pattern, as the patient experienced refractory rhinosinusitis before the appearance of yellow nail discoloration. Nail abnormality characteristics of YNS include thickened, over-curved nail plates, diminished longitudinal growth, scleronychia, and the absence of visible lunula and cuticle [1,7]. Hypotheses regarding the underlying pathogenesis suggest impaired lymphatic drainage and lipofuscin deposition as potential explanations for these nail findings [5].

Regarding respiratory involvement, chronic cough emerges as the prevailing symptom among YNS patients, with bronchiectasis and pleural effusions also frequently observed. Notably, pulmonary function testing in YNS patients without pleural effusions usually yields normal results [2]. Additionally, rhinosinusitis is a prevalent manifestation of YNS, affecting up to 83% of patients, with the maxillary sinus being the most commonly involved, followed by the ethmoid, frontal, and sphenoid sinuses. Nail changes may occur concurrently with, precede, or follow the onset of rhinosinusitis. Noncontrast sinus CT scans typically reveal mucosal thickening, occasionally accompanied by a fluid level [8].

YNS has been associated with various conditions, including autoimmune disorders, immunodeficiency, malignancy, nephrotic syndrome, hyperthyroidism, hypothyroidism, rheumatoid arthritis, tuberculosis, thiol drugs, and titanium implants [5,9]. The differential diagnosis of yellow nail discoloration is extensive, and all of the following potential causes should be considered when diagnosing YNS (Table 2).

**Table 2:** Differential diagnosis of patients with yellow nail discoloration Source: [5,10]

Drugs	Medical causes	Work-related causes
Quinacrine	Infection (onychomycosis)	Epoxy systems such as metaphenylenediamine
Topical 5-fluorouracil	Diabetes mellitus	Flower handling
Temsirolimus	Tobacco-associated use	Pesticides such as dinitroorthocresol
Bucillamine	Intense use of nail polish remover	Chromium salts
Retinoids	Lichen planus	Dyestuffs such as dinitrobenzene
Cetuximab	Psoriasis	-
D-Penicillamine	Chronic paronychia	
Tiopronin	Alopecia areata	
Gold	Onychogryphosis	
Methotrexate	Acquired pachyonychia	

The pathogenesis of YNS remains unclear, yet two hypotheses have been proposed. One theory suggests impaired lymphatic outflow, supported by findings from limb lymphoscintigraphy revealing low uptake percentages in the inguinofemoral and axillary lymph nodes among a series of six cases [11]. Moreover, higher uptake percentages were observed in control subjects compared to YNS patients, further supporting the impaired lymph transport hypothesis [12]. Conversely, Maldonado and Ryu proposed microvasculopathy with protein leakage as an alternative explanation for the clinical features observed in YNS [13].

Up to 30% of individuals with YNS experience spontaneous resolution in nail color without any specific treatment. Interestingly, this improvement appears to be more pronounced in fingernails than toenails, possibly due to the persistent lymphedema affecting the lower extremities [6]. In some cases, oral vitamin E and zinc administration have shown success in managing nail discoloration associated with YNS, reducing the yellowish hue. However, these treatments do not address the pulmonary manifestations of the syndrome [5,14]. Triazole antifungals have also demonstrated efficacy in treating nail changes, particularly when supplemented with oral vitamin E. Moreover, oral fluconazole, in combination with α-tocopherol, has induced clinical cure of nail discoloration in 85% of patients [15].

In our case, vitamin E, zinc, or antifungal treatment did not yield any improvement in the patient's nail condition, posing a challenge in management. Furthermore, multiple courses of antibiotics failed to resolve the patient's sinusitis symptoms or yellow nails. These findings provide additional evidence suggesting that the etiology of YNS in our patient is not mediated by chronic rhinosinusitis, indicating the presence of an underlying occult cause contributing to the illness.

The incidental identification of an odontogenic abscess and subsequent tooth extraction, resulting in gradual improvement in our patient's condition, underscore the significance of exploring minor causes when managing individuals with YNS, particularly in cases where classical lower respiratory tract involvement is absent. Furthermore, there are no reported cases where the treatment of chronic rhinosinusitis has led to clinical remission in patients with YNS. This observation emphasizes the notion that chronic rhinosinusitis is an associated manifestation rather than a contributing pathophysiological factor of YNS. To our knowledge, our case represents the first documented instance suggesting a tooth abscess as the underlying cause of YNS.

## Conclusions

This case clearly illustrates the triad of yellow nails, lymphedema, and respiratory tract involvement with YNS. The resolution of the patient’s symptoms following tooth extraction solidifies the association between the odontogenic abscess and the YNS. This case highlights the unusual presentation of a YNS associated with an odontogenic abscess. Our patient is also younger than the usual patient population reported to have YNS. Even though YNS is a rare condition with no clear etiology and no definite treatment, it is important to evaluate patients with this syndrome closely and thoroughly for secondary causes to avoid potential delays in management. Further research and larger trials are needed to gain a better understanding of the pathophysiology of YNS, its various presentations, and to help establish treatment guidelines.
